# Nutritive Importance and Therapeutics Uses of Three Different Varieties (*Murraya koenigii*, *Micromelum minutum*, and *Clausena indica*) of Curry Leaves: An Updated Review

**DOI:** 10.1155/2021/5523252

**Published:** 2021-10-31

**Authors:** D. T. Abeysinghe, D. D. D. H. Alwis, K. A. H. Kumara, U. G. Chandrika

**Affiliations:** ^1^Department of Chemistry, Faculty of Natural Sciences, The Open University of Sri Lanka, Nawala, Nugegoda, Sri Lanka; ^2^Department of Biochemistry, University of Sri Jayewardenepura, Nugegoda, Sri Lanka

## Abstract

*Murraya koenigii (M. koenigii)*, *Micromelum minutum (M. minutum), and Clausena indica* (*C. indica*) are three varieties of curry leaves in the family Rutaceae. They have been widely used in Ayurvedic medicine worldwide in the treatment and prevention of various diseases. Earlier findings provide strong evidence to support the three curry leaf species' potent pharmaceutical and biological effects, including antioxidant, antidiabetic, anti-inflammatory, and antitumor activities. Various parts of these plants, such as leaves, seeds, flowers, and fruit, contain constituents responsible for the modulation of numerous biological processes. Leading constituents of curry leaves play a crucial role in diabetic and anticancer management by regulating various molecular pathways, including Bcl-2, Bax, NF-*κ*B, and TNF*α*, according to *in vitro* and *in vivo* models established. Therefore, this review summarizes the current knowledge on research achievements made in terms of phytoconstituents, their structures, biological activities, and pharmacological actions with clinical studies of curry leaves up to date. The review also emphasizes the necessity for comprehensive research studies on the pharmacological actions and the mechanisms of selected phytochemicals of *M. koenigii, M. minutum*, and *C. indica* to validate their efficacy as potent herbal remedies for many ailments.

## 1. Introduction

The use of plant-based natural products in the treatment and prevention of diseases and health enhancement due to nutritive importance and pharmacological benefits has led to the significant attention of the scientific community and the general public nowadays. Medicinal plants are readily available and provide a cost-effective source with lesser side effects to develop new drugs [1, 2]. Plant-based traditional medicine is the stronghold of societies of many Asian countries in dealing with health and has a long history since ancient civilization, which uses plant materials as a significant ingredient in synthesizing drugs in different forms such as decoctions. It is a widely accepted fact that the rapid development of deriving pharmacologically active drugs from medicinal herbs has a tremendous impact on current medicinal practices [3]. For instance, in cancer treatment, more than 60% of all pharmaceuticals on the current market are natural products or mimics of natural products [4]. Furthermore, research has showed that plant-based diets rich with medicinal herbs are low-risk interventions that lower body mass index (BMI), blood pressure, glycated hemoglobin (HbA1C), and cholesterol levels. Therapeutic herbs may also reduce the number of medications needed to treat many metabolic and noncommunicable diseases, including diabetes, cancers, cardiovascular diseases, obesity, and the majority of metabolic and noncommunicable diseases are linked with higher mortality and morbidity rates [5]. Curry leaves are the most common herb used in Asia with enormous nutritive and pharmacological benefits. Therefore, this review is mainly focused on the phytochemical constituents and their bioactivities in three different varieties of curry leaves, *Murraya koenigii* (*M. koenigii*), *Micromelum minutum* (*M. minutum*), and *Clausena indica* (*C. indica*) available in most Asian countries. Their phytochemical constituents play a role in disease management through the managing of many metabolic pathways.

**Figure 1:**
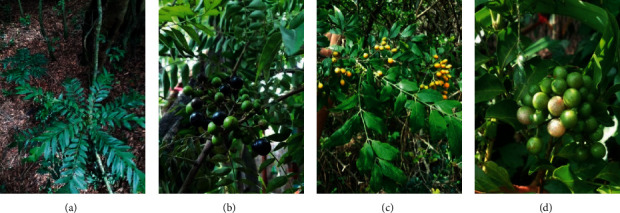
Morphologies of (a) leaves of *M. koenigii,* (b) fruits of *M. koenigii*. (c) Leaves and fruits of *M. minutum.* (d) Leaves and seeds of *C. indica*.

Rutaceae, the family of flowering plants, is composed of 160 genera and a few herbaceous perennials [6]. Of the 2070 global species belonging to the family Rutaceae, only a few herbaceous varieties are available in Sri Lanka, including *M. koenigii, M. minutum*, and *C. indica*. Of the three, *M. koenigii* is a fascinating house plant grown in Asia and native to Sri Lanka, Bangladesh, and India, which had used curry leaves or “karapincha” (in Sinhala) for centuries. The dark green fresh leaflets of *M. koenigii* are widely used in Asian cooking mainly for their aroma and versatile medicinal properties [7, 8]. Furthermore, they add subtle flavors to various food preparations, from vegetables to many other dishes as a natural flavor. Kola kanda or leafy porridge is famous in Sri Lanka for its high nutritional value. Various parts of *M. koenigii* are used to treat diabetes, chronic fever, dysentery, and diarrhea [9]. *M. minutum* is also used as a flavoring agent and is reported to have medicinal value in the southern part of Thailand and many other Asian countries [10]. In particular, *M. minutum* roots are used to cure ringworms and to regulate menstruation. Other parts of *M. minutum* are used as carminatives, purgatives, and expectorants [11, 12]. The leaves of *M. minutum* are used traditionally to treat toothache and teething issues in babies, skin irritations caused by scabies, and as a remedy for stomachache and headache [13]. *C. indica* is famous as a folk medicine in many Asian countries. Leaves and roots of *C. indica* treat various health issues, such as flu, colds, joint dislocation, bone fractures, headaches, colic, and rheumatism. Moreover, *C. indica* fruits are widely used in Vietnam and South Indian cooking mainly due to their aroma [14, 15]. The three varieties of curry leaf, *M. koenigii, M. minutum*, and *C. indica* discussed in the review, have somewhat common morphological characteristics and taxonomic positions as indicated in Table 1. Morphologies of the leaves and fruits of *M. koenigii*, *M. minutum, C. indica* are shown in Figure 1.

## 2. Beneficial Pharmacological Activities of *M. koenigii*, *M. minutum*, and *C. indica* and Their Isolated Constituents

**Figure 2:**
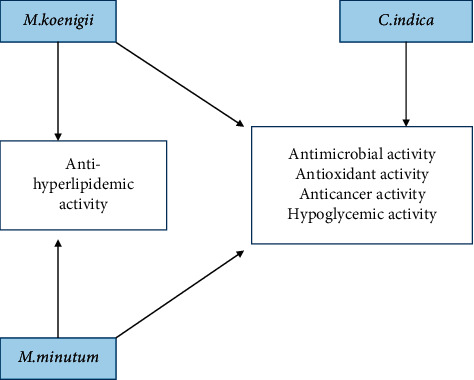
Major pharmacological activities of *M. koenigii*, *M. minutum,* and *C. indica*.

In the recent past, the use of *M. koenigii and M. minutum* in traditional medicines and home remedies has attracted the scientific community, but limited studies have been conducted to evaluate their pharmacological and medicinal efficacy. Despite the importance of *C. indica* in folkloric remedies, its pharmacological potential and chemical constituents have been rarely identified. Therefore, this review will shed light on the major bioactive compounds of *M. koenigii*, *M. minutum*, and *C. indica* and their pharmacological effects highlighting the potential in the key medicinal activities through the modulation of various biological pathways. The major pharmacological activities of the *M. koenigii*, *M. minutum*, and *C. indica* are discussed in detail in this review (Figure 2).

Several studies have contributed to understanding the chemical constituents and therapeutic actions of *M. koenigii, M. minutum*, and *C. indica*. Their bioactive compounds have been extracted from various solvents (water, hexane, dichloromethane, methanol, ethyl acetate, and benzene) and have been thoroughly explored using different techniques, including mass spectrometry, Nuclear Magnetic Resonance Spectroscopy (NMR), Infrared, and ultraviolet spectroscopy. Alongside these studies, pharmacological activities of the isolated compounds have been carried out via *in vitro* and *in vivo* studies combined with animal models.

Tables 2–4 summarize the major chemical constituents of *M. koenigii, M. minutum*, and *C. indica* and their pharmacological activities. All three varieties have nutritive value owing to the presence of a variety of essential phytochemicals, minerals, and trace minerals present primarily in their leaves and seeds. Phytochemical studies of the leaves, roots, seeds, and stem bark of *M. koenigii, M. minutum*, and *C. indica* have yielded many essential metabolites such as terpenoids, polyphenols, coumarins, alkaloids, flavonoids, carotenoids, vitamins, and nicotinic acid, as summarized in the supplementary information Tables S1–S3.

**Figure 3:**
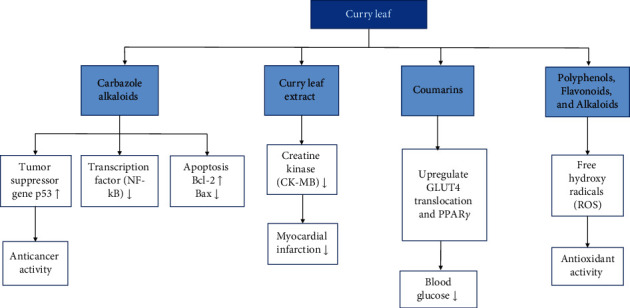
Modulation of different cell signaling pathways in disease management by the phytoconstituents in curry leaf.

The three species selected in this review, *M. koenigii, M. minutum*, and *C. indica*, have demonstrated a wide range of common pharmacological effects such as antihyperlipidemic, antimicrobial, antioxidant, and antihyperglycemic, and apoptotic activities [19, 32–35]. The two species, *M. koenigii and M. minutum*, have demonstrated remarkable cytotoxic anticarcinogenic activities. In addition, *M. minutum* has shown a remarkable effect as a poultice for ringworms [35]. *M. koenigii, M. minutum*, and *C. indica* contain various phytoconstituents that play an essential role in disease management via modulation of different cell signaling pathways (Figure 3).

### 2.1. Antihyperglycemic Activities

Diabetes mellitus is a deep-rooted disorder because the body cannot maintain blood glucose levels and needs long-term/lifetime medication [36]. Hyperglycemia or high blood glucose is a symptom of diabetes mellitus. In 1995, the estimated prevalence of diabetes among adults was 4%, and specialists predict that it will rise to 5.4% in 2025 [37]. For the management of diabetes mellitus, a particular remedy must have excellent glycemic control activity. If diabetes is left untreated, there is a possibility of degeneration even from its primary stages. It is associated with multiple disorders such as chronic hyperglycemia, weak carbohydrate, protein, and fat metabolism resulting from imperfections in insulin secretion. It was also found that life pattern changes can affect diabetes with considerable subsequent complications [36].

Etiologies of diabetes are highly variable. Two significant forms of diabetes exist, commonly referred to as type 1 and type 2. Among them, type 1 is recorded as a T cell-mediated autoimmune disease. The latter (type 2) diabetes is reported as 90% of the diabetes population in the world, being the most prevalent form of diabetes that is referred to as non-insulin-dependent diabetes. Type 2 diabetes may be owing to obesity and unhealthy lifestyles among individuals. While insulin production is present in this form, the extent of produced insulin may be inadequate to meet the body's needs or, in some cases, may be due to insulin resistance development [36]. Recently, investigators have studied the effect of curry leaves for the treatment of diabetes as they provide an affordable source with low side effects.

In this regard, a large amount of work has been done using animal models to study the effectiveness of *M. koenigii* leaf extracts on hyperglycemic activity. Rats have been selected as suitable animal models after inducing diabetes with different drugs, alloxan or streptozotocin (STZ), to mimic human hypoglycemic conditions. Several studies on the antihyperglycemic activity of *M. koenigii* in various solvents with varying degrees of polarities are reported. The rationale behind using solvents of varying polarity is to search for more promising drugs to manage hypoglycemic conditions. Water is the most common solvent used because of *M. koenigii's* common use in aqueous food preparations and decoctions.

A study with alloxan-induced diabetic rats administrated with the aqueous extract of *M. koenigii* leaves demonstrated a substantial decrease in the blood glucose levels of diabetic mice. Notably, no effect on the blood glucose levels of normal treated mice was observed, and the aqueous extract exhibited a significant reduction of the blood glucose level in rats at a dose of 200 mg/kg. This observation indicates a promising therapeutic effect on diabetes by *M. koenigii* with fewer or no undesirable side effects [37]. Several studies on aqueous leaf extracts are in line with this observation of the efficacy of *M. koenigii* in treating hyperglycemia [38–40].

Fresh juice of *M. koenigii* leaves has shown an effect on reduced blood glucose levels. Also, it enhances the effect of insulin by extending its therapeutic value in a study with healthy Wistar rats [41]. A successful combination of *M. koenigii* with insulin was achieved, and restoration of glucose homeostasis alone or in combination with other antihyperglycemic agents was suggested [41]. Therefore, the beneficiary effect in combination of *M. koenigii* with insulin indicates a clinical potential of developing a safe therapeutic approach to diabetic management that needs to be validated for the long run. A study was undertaken to evaluate the protective effects of *M. koenigii* leaves aqueous extract against beta-cell damage in streptozotocin-induced diabetes rats. The results showed that administration of *M. koenigii* leaves effectively brings about its antihyperglycemic effect *via* secretion of insulin secretion from the regenerated beta cells and remnant beta cells [40]. Another study was made to investigate the hypoglycemic activity of aqueous and methanolic *M. koenigii* leaves extracts on plasma insulin and blood glucose levels in alloxan-induced diabetic rats. These researchers had successfully achieved a significant reduction (*P* < 0.05) of blood glucose levels of diabetic rats treated with methanol and aqueous extracts compared to the diabetic control group. A significant rise in plasma insulin was observed on the 43^rd^ and 58^th^ days of treatment in methanol and aqueous extracts suggesting stimulated secretion of insulin from the beta cells of pancreatic cells [42]. Another study investigated the efficacy of feeding *M. koenigii* leaves as dietary constituents in controlling hyperglycemia in normal rats, alloxan- or STZ-induced mild diabetic rats, or moderate diabetic rats [43]. It was observed that the mild diabetic rats displayed a moderate antihyperglycemic activity but a poor antihyperglycemic activity was observed in moderately diabetic rats. However, the feeding of *M. koenigii* leaves did not affect any profound hypoglycemic effect in normal rats, as stated in previous findings [43]. Another study was made to evaluate the hypoglycemic effect of ethanolic leaf extracts of *M. koenigii* on alloxan-induced diabetic rats, where results revealed that the extract exhibited hypoglycemic effects [44]. Another study revealed that the chloroform extract of *M. koenigii* enhanced insulin secretion from the pancreas [45].

Raised glycated hemoglobin concentration in plasma is the clinical hallmark of poorly controlled diabetes [46]. Glycated hemoglobin is a modified hemoglobin product of a nonenzymatic condensation of glucose with hemoglobin during hyperglycemia. Therefore, glycated hemoglobin (HbA1C) contributes as a biomarker, easily indicating the management status of diabetes in patients [47]. Studies have been carried out to determine the effect of *M. koenigii* on hemoglobin glycation and alloxan-induced diabetic rats with a blood glucose level of 13 mmol/l. Alloxan-induced diabetic rats were treated orally with a crude aqueous extract (500 mg/kg body weight), and the outcome of the study indicated a significant (*P* < 0.05) decrease in blood glucose and glycated hemoglobin levels compared to the diabetes control group [47].

Inhibition of hemoglobin glycations is considered to be one of the therapeutic approaches that delay or prevent the progression of major diabetic complications. A method was developed with bovine serum albumin (BSA) using polyacrylamide gel electrophoresis under native conditions (PAGE) to investigate the *in vitro* protein glycation inhibitory potential of *M. koenigii* aqueous leaf extract. The results revealed that, in the presence of *M. koenigii* aqueous leaf extract, migration of BSA under nondenaturing conditions was retarded compared to the control [48]. This work suggested an *in vitro* protein glycation inhibitory potential of the *M. koenigii* aqueous leaf extract.

Diabetic peripheral neuropathy, a peripheral nerve disorder, develops as a late manifestation of long-standing or uncontrolled diabetes. A study was made to evaluate neuropathy management in male Wistar rats by the ethanolic extract of *M. koenigii* leaves. The study results showed that ethanolic extract successfully regulated the blood glucose level and managed neuropathy [49]. Studies of this manner possibly pave the way for more natural drugs in treating diabetes and its related disorders.

The loss of pain perception occurs in diabetic patients due to damage of nerves. An experiment was made to investigate the pain threshold of the streptozotocin-induced diabetic rats after administering a dose of 300 and 500 mg/kg ethanolic extract of *M. koenigii*. The increased latency time in streptozotocin-induced diabetic rats suggested pain perception in the tested animal model. Therefore, the ethanolic extract of *M. koenigii* significantly decreased the glycemic level and prevented the progression of neuropathy in the diabetic animal model [49]. Furthermore, among hundreds of plants that have been studied for diabetes, *M. koenigii* is under clinical trials. One clinical research indicated a transient reduction in fasting and postprandial blood sugar levels in non-insulin-dependent diabetes mellitus patients provided with 12 g of *M. koenigii* leaves powder supplementation [50]. Among a wide range of phytochemical compounds isolated from *M. koenigii*, only a few compounds have been investigated on their antidiabetic potential. A study reported an *in vitro* antidiabetic activity in L6-GLUT4myc myotubes and *in vivo* studies of streptozotocin-induced diabetic rats with six major carbazole alkaloids, koenidine, murrayazoline, koenimbine A, koenidine, mahanimbine, and O-methylmurrayamine, extracted from *M. koenigii* leaves. The results revealed the potential use of koenidine in managing type II diabetes with insulin resistance [51].

The effect of *M. minutum* seeds ethanolic extract with coumarin and microminutinin has been shown to lower serum glucose levels in diabetic rats. A significant decrease in glucose levels was observed after administering coumarin (100 mg/kg) and microminutinin (25 mg/kg). The *in vivo* study further demonstrated the potential of using *M. minutum* seeds in treating hyperglycemia [13]. Similarly, the development of antidiabetic agents from *C. indica* roots has been investigated by bioguided isolation of the ethyl acetate extract. The isolates, dentatin, clausine K, and nordentatin, demonstrate anti-*α*-amylase activity suggesting the hypoglycemic activity of *C. indica* roots [52]. A recent study with ethyl acetate and hexane extracts of *C. indica* fruit identified coumarins as major compounds demonstrating anti-*α*-amylase activity [53]. Therefore, *C. indica* and *M. minutum*, too, could be promising sources of antidiabetic agents.

The active compounds of *M. koenigii*, *C. indica*, and *M. minutum* play a pivotal role in preventing and treating diabetes mellitus. Even though the exact molecular mechanism in this vista is not understood fully, it was considered that their constituents play a vital role in the modulation of various cell signaling pathways, including insulin secretion. Many of the experiments discussed above are attributed to the presence of coumarins, carbazole alkaloids, and their derivatives in the *M. koenigii*, *C. indica*, and *M. minutum* extracts, as they are considered to be potent antihyperglycemic agents [54]. Carbazole alkaloids such as mahanimbine and koenimbin can increase peripheral glucose uptake. The phytochemicals in the extracts protect the pancreatic islets and the *ß* cells, in addition to the enhanced secretion and sensitivity of insulin from the pancreas, as suggested by various studies. Umbelliferone and osthole are the most common coumarins studied to modulate molecular pathways in diabetes and its complications [54].

Peroxisome proliferator-activated receptor *γ* (PPAR*γ*) is an essential nuclear hormone receptor acting on the transcription of genes (e.g., glucose transporter gene GLUT4) involved in glucose disposal. A study finding reveals that umbelliferone upregulated the expression of surface GLUT4 gene and PPAR *γ* in adipose tissue, enhancing insulin sensitivity leading to increased peripheral glucose uptake [55]. Osthole also activated PPAR *γ*, increasing insulin sensitivity, and facilitated the synthesis and storage of fat [56]. The activation of these PPARs may also suppress inflammation. Umbelliferone further helps cells resist oxidative stress by facilitating the biosynthesis of enzymes responsible for radical attacks such as superoxide dismutase (SOD) and glutathione (GSH) [57]. In another perspective, umbelliferone inhibited the hydroxyproline level reducing the production of misfolded collagen in helping to treat collagen-induced diabetic nephropathy [54].

Keeping in view the tremendous antidiabetic studies and availability of literature, *M. koenigii* represents a promising candidate with potential use for the management of diabetes mellitus. Therefore, *M. koenigii* represents the clinical potential of developing a safe therapeutic drug confirmed with further preclinical and clinical research. Although plenty of evidence demonstrates that coumarins and other metabolites are effective against hyperglycemia, there had been no adequate investigations reported on the involvement of *C. indica* and *M. minutum* in hyperglycemia.

### 2.2. Anticancer and Cytotoxic Activities

Cancer involves multiple genetic alterations, unlike other monogenic disorders. The onset of cancer typically consists of a series of mutations in the genome resulting in uncontrolled cell proliferation, lack of apoptosis, and alterations in epigenetic regulations. Nearly ten million new cancers are diagnosed annually, making it the second most common cause of mortality worldwide [58]. Typical treatment strategies for most cancers include radiation therapy, hormone therapy, chemotherapy, and surgical tumor resections. Despite the drawbacks, including multidrug resistance in cancer cells and nonspecific targeting, chemotherapy remains the predominantly used treatment in many cancers. However, growing interest in using natural compounds paves the way for new perspectives in chemotherapy studies. *M. koenigii* and *M. minutum* have been reported to have a potential role as a remedy for cancer and inflammation. The preliminary approach for any potent anticancer compound from natural sources is an investigation of *in vitro* cytotoxicity studies on various cell lines. Numerous studies have been carried out on isolating natural compounds and understanding their antitumor and cytotoxic activities. *M. koenigii and M. minutum* also have been successfully addressed in this regard.

Constituents *M. koenigii*, *C. indica*, and *M. minutum* play an essential role in the modulation of various signaling pathways in the cell. These constituents upregulate the tumor suppressor genes *p*53 and downregulate the genes responsible for developing cancer, such as NF-*κ*B. Furthermore, various carbazole alkaloids and coumarins of these curry leaves activate the cyclooxygenase pathway and apoptosis.

#### 2.2.1. Effect of Curry Leaves and Their Constituents on Cytotoxicity

Some chemotherapeutic drugs have proved to be highly cytotoxic agents to normal cells, as seen in clinical studies. However, the preferential cytotoxicity against malignant tissues is crucial in a suitable treatment. Plants and their derivatives play an essential role in the cytotoxicity studies searching for an alternative for chemotherapeutic drugs with selected toxicity towards cancerous cells. Active compounds in a plant extract target cell lines and significantly affect cytotoxicity.

An experimental study reported the cytotoxic activities of alkaloid extract from *M. koenigii* in the breast cancer cell line MDA-MB-231 with an IC_50_ of 14.4 *μ*g/mL [59]. Mahanine, a carbazole alkaloid isolated from *M. koenigii* and *M. minutum,* and its analogs have shown significant cytotoxic and anticancer activities in prostate cancer studies. Prostate cancer, the most commonly diagnosed cancer in men worldwide, is associated with alterations in androgen receptor functions. Studies have shown the functions of mahanine related to the androgen receptor, inhibiting ligand-dependent and -independent transactivation leading to a significant decrease in the expression of androgen-regulated genes. Furthermore, mahanine results in proteasomal degradation by compromising the stability of the androgen receptor [60]. Another study with a combination of mahanine and cisplatin, a drug used in cervical cancer treatments, reported cytotoxicity even at reduced concentrations [61]. The dual combination of mahanine and cisplatin inhibited cancer cell migration and was further suggested to be a prospective combination for cancer treatment due to the lower toxicity reported.

In another study, mahanine isolated from *M. minutum* executed apoptosis through the intrinsic pathway in human leukemia U937 cells [62]. A study with *M. minutum* leaves methanol extract composed of six coumarins including minutin A, minutin B, 8,4″-dihydroxy-3″,4″-dihydrocapnolactone-2′,3′-diol, 8-hydroxyisocapnolactone-2′,3′-diol, 8-hydroxy-3″,4″-dihydrocapnolactone-2′,3′-diol, and clauslactone E reported a significant cytotoxic activity against lung adenocarcinoma (A549 and SBC3) and leukemia (K562 and K562/ADM) cell lines [27]. Furthermore, 8-hydroxyisocapnolactone-2′,3′-diol displayed significant cytotoxic activity against both cervical cancer (HeLa) and liver cancer (HepG2) cell lines suggesting potential nutraceutical chemopreventive agents from *M. minutum* leaves.

Specific cytotoxicity activities of the extracts of *M. minutum* roots and its isolates were reported to have inhibitory effects against the KB cell line and weak cytotoxicity against the NCI-H187 cell line providing a basis for the use of *M. minutum* roots in the treatment of cancers [28]. The various preclinical human carcinoma models are to be further investigated on *in vivo* efficacy to promote various isolates of *M. koenigii* and *M. minutum* to be used in treatments in carcinoma.

#### 2.2.2. Effect of Curry Leaves and Their Constituents on Apoptosis

Apoptosis is a typical biochemical function that induces the committing of cell death of pointless cells. Resistance towards apoptosis is a crucial factor of tumor cells. Therefore, any search for the new chemotherapeutic agent that can induce apoptosis is enhanced by understanding apoptotic pathways. The two main pathways of apoptosis are intrinsic and extrinsic. External death signals trigger apoptosis in extrinsic pathways, and intrinsic pathways are mediated by Bcl-2-associated X protein (Bax) and Bcl-2 antagonist. Any alteration in Bcl-2 and Bax causes the development of cancers [63].

Several investigations provided evidence of the proapoptosis functions of the *M. koenigii-* and *M. minutum-*derived extracts and phytochemicals, including carbazole alkaloids such as mahanine, mahanimbicine, girinimbine, mahanimbine, and coumarins. A valuable study on the effect of mahanine in prostate cancer reported the induction of apoptosis in cancer cells. Several bioactive compounds, including girinimbine, a carbazole alkaloid isolated from *M. koenigii,* have been reported to potentially inhibit inflammation and induce apoptosis in human colon cancer cells (HT-29) *in vitro* [64]. The induction of apoptosis by girinimbine was also evidenced *in vivo* in a 24-hour treatment of zebrafish embryos, indicating a significant distribution of apoptotic cells in embryos. These findings strongly suggest a potential for girinimbine to be further examined for its use in treating cancer as a chemotherapeutic agent. Another *in vitro* study of girinimbine on A549 lung cancer cells about apoptotic mechanistic pathways has demonstrated a successful induction of the early phase of apoptosis as indicated by stability assays [65]. Furthermore, the use of girinimbine and its derivatives as a herbal remedy in combinational chemotherapeutics have been recommended in several other *in vitro* and *in vivo* studies [65, 66].

These findings strongly represent the notion that girinimbine has a significant involvement in both intrinsic and extrinsic pathways of apoptosis. Moreover, girinimbine upregulates the p53 the key tumor suppressor protein, and the cyclin-dependent kinase (CDK) proteins, p27 and p21. The overexpression of CDK proteins inhibits abnormal cancer cell proliferation and promotes cell cycle arrest at the G1 phase. Additionally, girinimbine activated apoptosis *via* regulating the intrinsic pathway by the downregulated Bcl-2 protein expression and upregulated Bax [27, 63]. Another study revealed that girinimbine-treated HepG2 cells ultimately led to apoptosis through G_0_/G_1_ phase arrest [67]. In this regard, a large amount of work has been done with girinimbine to study its effectiveness on diverse cancer types.

Koenimbine, another dietary component isolated from *M. koenigii,* has been studied to evaluate the efficacy of the inhibition of MCF7 breast cancer cells through apoptosis [68]. This study adds insight into the ability of koenimbine to trigger apoptosis in breast cancer cells *in vitro*. One possible mechanism of koenimbine in apoptosis in breast cancer cells could be the downregulation of the Wnt/*β*-catenin self-renewal pathways. Similarly, koenimbine has remarkably inhibited cell proliferation of prostate cancer stem cells in a recent study [69].

In a previous study, *C. indica* roots were used to purify few compounds, including dentatin, nordentatin, and clausine K, with anticarcinogenic properties [52]. In addition, *C. indica* is reported to be a rich source of phytochemical components such as sesquiterpenes, coumarins, and carbazole alkaloids which might have a strong potential for anticancer activity. Nevertheless, there is still room for studies on *C. indica*, which needs to be screened for the novel, cost-effective, and efficacious anticancer agents.

#### 2.2.3. Effect of Curry Leaves on NF-*κ*b Transcription Factor

The transcription factor, NF-*κ*B transcription factor, has a pivotal role in human cancers. The overexpression of NF-*κ*B induces antiapoptosis and angiogenesis and promotes tumors. Several stimuli such as cellular and environmental stresses, DNA damage, growth factors, and cytokines activate NF-*κ*B. Therefore, the inhibition of NF-*κ*B is crucial in the prevention and control of cancer. A study evaluated the chemopreventive activity of *M. koenigii* leaf extract on 4T1 breast cancer cell-challenged mice. The results confirmed those aqueous leaf extracts significantly prevented tumor formation compared to the untreated group of mice. The activation of NF-*κ*B is induced by the cytokines cytokineIL-1*β* and pleitropic cytokine IL-6 was found to be reduced in the *M. koenigii-*treated 4T1 breast cancer cell-challenged mice [70].

### 2.3. Effect on Cardiovascular Diseases

Cardiovascular diseases are the number one cause of death in the world. According to WHO, cardiovascular diseases in the elderly population have created 18 million deaths in the year 2016, resulting in 31% of deaths in the globe [71]. Atherosclerosis is one of the major causative factors for cardiovascular disease due to the blockages that prevent blood from flowing to the heart or brain due to the building up of fatty deposits on the inner walls of the blood vessels. Abnormally increased levels of lipids in the blood, triglycerides, phospholipids, cholesterol, and cholesterol esters trigger atherosclerosis. The different types of cholesterol, High-Density Lipoprotein (HDL), Low-Density Lipoprotein (LDL), and Very Low-Density Lipoprotein VLDL, have a massive impact on the prevalence of cardiovascular disease.

Various studies are undertaken to evaluate the antihyperlipidemic potential of the *M. koenigii* leaf extracts. The biochemical response of *M. koenigii* was studied on various types of cholesterols in a study with Albino rats. These researchers fed Albino rats a diet fortified with 20% coconut oil with 10% *M. koenigii* leaf feed for three months. An increase in HDL and reduced LDL, VLDL, and total serum cholesterol levels were observed in the experiment [72]. In another study, the effect of *M. koenigii* aqueous leaf extract of 300 mg/kg on the biochemical response in normal and STZ-induced severe diabetic rats were evaluated. The results confirmed a decrease of 22.9% and 37.1% of triglyceride level and 19.2% and 30.3% in total cholesterol in normal and diabetic rats, respectively, in one month [39].

The potency of *M. koenigii* leaf on lowering blood cholesterol levels could be ascertained by various experimental conditions, including the study's duration, the type of animal model or the form of *M. koenigii* leaf used, or the solvent used for the extraction. Another finding showed that obese diabetic ob/ob mice given 80 mg/kg aqueous *M. koenigii* extract for ten consecutive days have significantly decreased blood cholesterol levels from 277.6 ± 16.6 mg/day (day 0) to 182.0 ± 15.3 mg/day [73]. In addition, the dichloromethane and ethyl acetate extracts of *M. koenigii* leaves lowered body weight gain in high-fat diet-induced obese rats at oral administration of 30 mg/kg/day.

Creatine kinase (CK-MB) is a well-known heart enzyme and a cardiac biomarker found primarily in the cardiac muscle. A study assessed the effect of *M. koenigii* aqueous extract (180 mg/kg body weight) on albino Wistar rats treated with Voltral tablets to increase the level of CK-MB [74]. A significant decrease of the heart enzyme CK-MB was observed in rats treated with *M. koenigii* compared to the group treated with atenolol, a drug available in the market as a beta blocker, further suggesting a potential role of *M. koenigii* in lowering CK-MB in myocardial infarction.

*M. minutum* seed, a rich source of coumarins [29], has been used to investigate the efficacy of the variation of serum cholesterol, triglycerides, HDL, and LDL levels in diabetic rats [13]. In that study, a significantly increased HDL was observed with the administration of 100 mg/kg ethanolic extract of *M. minutum* seeds in rats. However, no report on the potency of *C. indica* on treating or reducing cardiovascular biomarkers was found in the literature.

### 2.4. Antimicrobial Activity

The occurrence of microbial drug resistance of many microorganisms and their complications are growing worldwide. Therefore, antibiotic resistance has become one of the major issues since the late 20^th^ century's rising demand for new antimicrobial agents. However, most new antimicrobial agents fail after many clinical trials and treatments due to the side effects and rapid resistance development. Traditionally, *M. koenigii*, *M. minutum*, and *C. indica* are well known for their health benefits and are rich in antimicrobial activities owing to the presence of a variety of essential phytochemicals and many secondary plant metabolites, such as tannins, terpenoids, alkaloids, flavonoids, phenols, and quinines present in their leaves and seeds [75].

The biodeterioration of food due to microorganisms causes a significant loss to crop from vegetables, grains to fish, and poultry. Many plant extracts have been reported to display antifungal, antibacterial, and insecticidal properties *in vitro*. Therefore, aqueous extracts of widely used culinary spices, including *M. koenigii,* were screened in a research study against several microorganisms found on edible fish to identify their antimicrobial activities [76]. *M. koenigii* has shown a significant antibacterial activity towards various pathogenic bacterium types found in edible fishes. The minimum inhibitory concentration (MIC) of *M. koenigii* on *Staphylococcus aureus (S. aureus)* was 250 *μ*g/ml and 500 *μ*g/ml on *Escherichia. coli (E. coli)*, *Lactobacillus* spp*., Salmonella typhi (S. typhi),* and *Shigella spp*. Both methanol and aqueous extracts of *M. koenigii* showed antibacterial activity among all the herbs extracts tested in the study suggesting the use of *M. koenigii* in cooking to eradicate the respective bacteria and as a food preservative.

An investigation was carried out to screen twenty medicinal plant extracts against seed-borne fungi in seeds, causing weight and quality losses leading to the reduction of commercial value. This study was aiming at the efficacy of *M. koenigii* on seed-borne fungi species. *M. koenigii* was reported to have a remarkable mycelial growth inhibitory effect against all the seed-borne fungi used in the experiment. However, the highest inhibition of mycelial growth was observed in *Alternaria alternata* [77]. In view of these, MICs of bactericidal and fungicidal activities were reported for methanolic, ethanolic, and acetonic leaf extracts of *M. koenigii* in a study [78]. Acetonic and methanolic extracts showed the highest susceptibility for the bacterial species tested, indicating the role of the extraction efficiency of bioactive compounds in different solvents.

In evaluating the antioxidant property of *M. koenigii* plant parts, bark extract is the least common among researchers. Few investigators have used the agar cup-plate method in accessing the antibacterial activity of different microbial species of the bark and leaf extracts. Bark extracts have shown a significant inhibitory activity compared to the leaf extracts of *M. koenigii,* and the most susceptible species was *S. aureus* [33]. Leaf and seed extracts of *M. koenigii* in different solvents, acetone, chloroform, ethanol, and methanol, have been studied for their antimicrobial activities. The study results indicated that the acetone and methanol extracts had higher antimicrobial activity than other solvents [79].

Another study revealed the *in vitro* antibacterial potential of the aqueous and ethanolic leaf extracts of *M. koenigii* against bacterial species accountable for endometritis. *Bacillus* sp*., Salmonella* sp*., Staphylococcus* sp*.,* and *Corynebacterium* spp. are responsible for endometritis in domestic cows. Ethanol and aqueous extracts of *M. koenigii* showed a potent antimicrobial effect towards pathogens under study. The ethanolic extract showed a better response than the aqueous extract [80].

Essential oils extracted from plants have been recognized for their antioxidant and antimicrobial attributes for decades. Essential oils of *M. koenigii* leaves are rich in many phytochemicals, serving as antioxidant and antimicrobial agents [81]. The antimicrobial activities of essential oils from *M. koenigii* have been extensively explored recently. An experiment was made to evaluate the antifungal activity of methanol and ethanol extracts and the essential oils of *M. koenigii* leaves. The results indicated that all the extracts showed antifungal activity and methanol extract had the highest activity and essential oils with a moderate antifungal activity [79].

*M. minutum is* reported to be a rich source of antimicrobial compounds. Antibacterial potential of leaves, stem bark, and root bark ethanolic extracts of *M. minutum* against *E. coli* and *S. aureus* was investigated to validate its current use as alternative medicine in a study [82]. The inhibitory activities of the three ethanolic extracts towards *S. aureus* were significant when compared with *E. coli.* Among the three extracts, leaf extracts showed the highest zone of inhibition towards both bacteria species due to the prevalence of highly cytotoxic coumarin compounds and highly antimutagenic carbazole alkaloids. For instance, mahanine could explain the increased inhibitory activity of leaf extracts [82]. However, stem bark and root extracts of *M. minutum* showed partial activity towards *S. aureus* and inactivity towards *E. coli* in this study.

The essential oil from *M. minutum* is rich in sesquiterpenes germacrene, germacrene-B, *δ*-elemene, and *ß*-caryophyllene, having a potential antimicrobial activity [83]. The antimicrobial activity of *M. minutum* essential oils was investigated at a concentration of 0.25%—1% (v/v) in a study. The results showed significant antimicrobial activity at 1% v/v towards all the species under study [30].

A study was performed on the antibacterial activity of *C. indica* leaf essential oil extracts from South India, and significant activity against *B. cereus, S. typhi,* and *P. vulgaris* was observed [14]. However, no significant activity was reported against *E. coli* and *S. aureus.* Another experiment was made to evaluate the MIC of essential oils from *C. indica* from Vietnam towards selected Gram-negative and Gram-positive bacteria. The results showed significant antibacterial activity against *E. coli* and *S. aureus*. In addition, the MIC of various fungi strains was also tested, and interestingly, the essential oil was inactive for all the tested fungi species [14]. Therefore, the results of antibacterial assay for *E. coli* and *S. aureus* are contradictory in the studies conducted in South India and Vietnam, signifying a difference in their chemical profiles. The essential oil from *C. indica* from Vietnam leaf contains terpenes, mainly terpinolene, and myristicin, as the major constituents [19]. At the same time, sabinene was the major component in the same species from South India [14].

Hardly any literature was found on the antimicrobial activity of *M. koenigii* or *M. minutum* fruits, but the antimicrobial activity of *C. indica* fruits has recently been reported [84]. Flavonoids, a significant constituent in the fruits of *C. indica,* have shown significant antimicrobial effects against the Gram-positive bacteria, and no activity was reported for the tested fungi and yeast species [84]. Microorganisms tested for the antimicrobial potential of *M. koenigii, M. minutum,* and *C. indica* observed in literature are tabulated in Table 5.

### 2.5. Antioxidant Properties

Medicinal plants are excellent natural antioxidants that can be used to prevent diseases such as cancer, heart diseases, strokes, and inflammation [94]. The secondary metabolites, phenolics, flavonoids, and carotenoids are found in almost all parts of medicinal plants, and they have been reported to have potent free radical scavengers and antioxidant properties [95]. The growing interest in antioxidants from natural sources has attracted the scientific community recently, as synthetic antioxidants have several side effects such as the risk of liver damage and carcinogenesis in animals [96, 97].

*M. koenigii* plays a vital role among the traditionally important medicinal plants utilized in Asian ayurvedic practices due to their health benefits and therapeutic actions [95]. Various direct and indirect methods have assessed the antioxidant activities of the plants of the Rutaceae family. Most parts of *M. koenigii* are reported to be a rich source of plant polyphenols, flavonoids, and alkaloids, possessing a high antioxidant potency [98–100]. A recent study reported that total antioxidant activity was the highest in *M. koenigii* (2691 *μ*mol of ascorbic acid/g sample) among green leafy vegetables [101]. The *in vitro* antioxidant property of leaf extracts of *M. koenigii* has been studied in different solvents/solvent mixtures such as water, methanol, hexane, water : ethanol, or chloroform with DPPH, Fe^3+^ ion reducing method, and hydroxy radical scavenging activity [102]. All samples varied in their antioxidant potential depending on the chemical composition and the solvent. Nevertheless, the 1 : 1 (water : ethanol) mixture leaf extract exhibited the maximum antioxidant activity. Interestingly, the 1 : 1 (water : ethanol) mixture was able to scavenge approximately 90% of DPPH and hydroxyl radicals at 4–5-fold lower concentrations than all the other tested solvent extracts. The observation of water: ethanol mixture leaf extract with the highest scavenging activity is in accordance with a study that used response surface methodology to optimize the solvent concentration and extraction time in maximizing the yield of *M. koenigii* leaf extracts to determine the total phenol content and DPPH assay. The 50% ethanol extract showed the highest scavenging ability and total phenolic content [103].

The H_2_O_2_ scavenging method has been used to screen the total antioxidant activity of *M. koenigii* acetone and petroleum ether leaf extracts. The mature leaf acetone extracts had a relatively greater scavenging activity by 50% compared to young leaves in petroleum ether [104]. A study was made to access the antioxidant activity of *M. koenigii* leaves in different solvent extracts, benzene, ethyl acetate, petroleum ether, acetone, methanol, and ethanol. It was evident that the benzene layer had the highest radical scavenging power, followed by ethyl acetate and petroleum ether [105]. This observation is further supported in another study, where the benzene fraction was found to have the highest total phenolic content and antioxidant activities [106]. Differences in extractability of the antioxidant components in a solvent with various polarities explain the varied antioxidant activities of *M. koenigii* in different solvents.

Another study was made to investigate the efficacy of *M. koenigii* powder as an antioxidant in in-ground goat meat and precooked goat meat. The results confirmed that the *M. koenigii* powder at 0.2% (w/w) acts as an effective inhibitor for oxidation products formed during raw ground and precooked meat during storage [99]. Many other studies have reported the antioxidant potential of the *M. koenigii* plant as a whole or various isolates from its bark, leaves, roots, or fruits with various methods and solvents, suggesting the pronounced antioxidant activity of *M. koenigii* [24, 85, 105].

An important study was made based on evaluating the antioxidant activity of *M. minutum* bark extract and the active fractions isolated in methanol fraction by preparative TLC method. *M. minutum* exhibited significant antioxidant activity with IC_50_ values of 54.3 and 168.9 *μ*g/mL, respectively [107]. The identification of hydramicromelinin in the active fractions was made based on detail spectral data as a potential lead of an antioxidant compound.

The potential antioxidant activity of methanol, hexane, ethyl acetate, and water extracts of *C. indica* fruits was evaluated. The results have showed that the highest total phenolic content was observed in the methanol extract [53]. Furthermore, methanol extract exhibited a remarkable antioxidant activity for DPPH and ABTS assays with the lowest IC_50_ values, 0.12 and 0.26 mg/mL, respectively.

Most of the isolated antioxidant active components in plant extracts of various solvents have been identified; antioxidant activities are diverse. These differences might be due to the extraction of curry leaves in a single solvent instead of fractionating them into different solvents. Furthermore, the various extraction methods and the extraction time and temperatures employed might have different levels and compositions of antioxidant compounds extracted, which further leads to the need for phytochemical characterization.

## 3. Conclusion

In recent years, the exponential growth of plants' use in medicine has been observed due to their remarkable pharmacological activities. The present review identified three of such important plants, *M. koenigii*, *M. minutum*, and *C. indica*, which have rich sources of bioactive metabolites. Their natural origin, low cost, and minimal adverse effects have attracted the scientific community to develop nutraceuticals and plant-based drugs. Clinical studies confirmed the pivotal role of *M. koenigii* and *M. minutum* in the prevention of various diseases. The amass quantity of information that has been accumulated in this regard has allowed us to understand the molecular mechanisms underlying these activities fully or partially in potential treatments. Although *C. indica* has been proven to be medicinally important, least or no attention was received by scientists. Therefore, further studies are essential for elucidating the role of the individual phytochemicals of *C. indica* in eliciting various pharmacological effects. The pharmacological effects of these three species differ on the part of the plant or fruit used. Furthermore, the geographical, seasonal, and other variations among countries and regions also influence the chemical composition. In closing, *M. koenigii*, *M. minutum*, and *C. indica* have proven to be promising candidates in both the drug designing by natural sources and the development of nutraceuticals. We believe that the current review provides a cumulation of information that will shed light innovatively for future investigation on the use of underutilized plant materials to discover new drugs with the aid of animal models and clinical trials.

## Figures and Tables

**Table 1:** Morphological parameters and taxonomic positions of *M. koenigii*, *M. minutum*, and *C. indica.*

Morphological parameters	*M. koenigii*	*M. minutum*	*C. indica*
Tree	Shrub or tree 6 m in height and 15–40 cm in diameter of the trunk	Shrub to small tree to 9 m high	Shrub to small tree
Bark	Grey color bark with longitudinal striations and white bark is present beneath it	Grayish brown bark	Grayish bark with scattered round glands to 0.35 mm diameter
Leaf	Leaves are bipinnately compound, 15–30 cm long, bearing 11–25 leaflets alternate on rachis, irregular margins	Leaves are pinnate with 7–15 leaflets. Each leaflet is 2–10 cm long, 1–6 cm broad	Compound, exstipulate, imparipinnate, large, 1–30 cm long; leaflets 7–13, shortly stalked, 3.7–8 cm long, unequal at base
Flowers	Bisexual, white, sweetly scented, stalked funnel-shaped, complete, diameter 1.12 cm terminal cymes each bearing 60–90 flowers	Petals are valvate. Cotyledons crumpled; style as long as ovary and white flowers in mostly flat-topped corymbose cymes	Inflorescence racemose –paniculate with greenish-white flowers, 6–8 mm long. Style shorter than the ovary
Fruits	Ovoid to subglobose, wrinkled, or rough with glands; 2.5 cm long and 0.3 cm in diameter purplish-black color when ripen; biseeded	Young fruits green, ellipsoid, and oblong, scented. A ripe fruit grows up to 5–9 mm long and appears in yellow, orange, and red color according to maturity	Globular berry, pink, or cream-colored, 1-2 cm long, gland-dotted
Seeds	Spinach green color, 11 mm long, 8 mm in diameter and weighs up to 445 mg [16]	Seeds with green and crumpled cotyledons	Solitary, green color
Taxonomy	Kingdom Plantae	Kingdom Plantae	Kingdom Plantae
	Subkingdom Tracheobionta	Subkingdom Tracheobionta	Subkingdom Tracheobionta
	Superdivision Spermatophyta	Superdivision- Spermatophyta	Superdivision Spermatophyta
	Division Magnoliophyta	Division Magnoliophyta	Division Magnoliophyta
	Class Magnoliopsida	Class Magnoliopsida	Class Magnoliopsida
	Subclass Rosidae	Subclass Rosidae	Subclass Rosidae
	Order Sapindales	Order Sapindales	Order Sapindales
	Family Rutaceae	Family Rutaceae	Family Rutaceae
	Genus Murraya	Genus *Micromelum*	Genus *Clausena*
	Species *Murraya koenigii* L. Spreng [17].	Species *Micromelum minutum* [18]	Species *Clausena indica* [19]

**Table 2:** The major bioactive compounds of *M. koenigii* and their pharmacological activities.

No	Plant part	Chemical type	Chemical	Structure	Activity	Ref
01	Stem	Alkaloids	Girinimbine	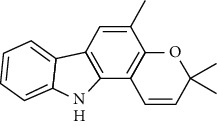	Antimicrobial/anticancer/antitumor	[9]
02	Leaves/stem		Murrayanine	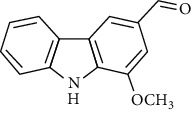	Antimicrobial/anticancer/antioxidant	
03	Leaves/stem		Mahanine	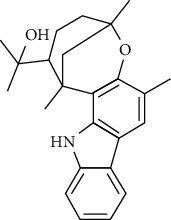		[7]
04	Stem/bark		Murrayacine	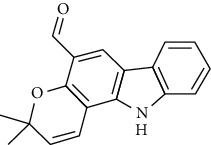		
05	Leaves		Murrayanol	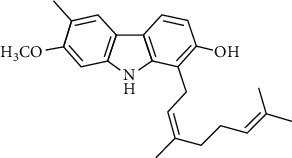	Antioxidant/anti-inflammatory/antimicrobial	[20]
06	Stem/bark		Mukoeic acid	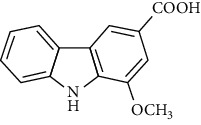	Antioxidant	[9]
07	Stem/bark		Murrayazolinine	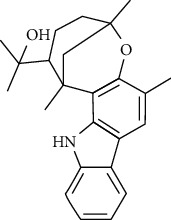	Anticancer	[9, 16, 21]

08	Roots	Alkaloids	Mukoline	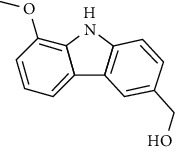	Cytotoxic/antimicrobial	[7, 9, 21]
09	Leaves/stem/bark	Alkaloids	Mahanimbine	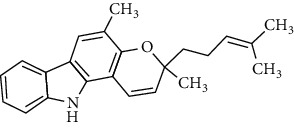	Antioxidant/anticancer	[7]
10	Leaves/bark		Koenine	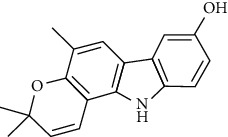	Antioxidant/antidiarrheal	[9]
11	Leaves		Koenigine	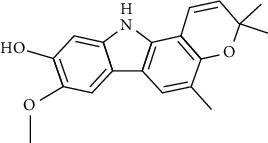	Antioxidant	[22]

12	Stem/roots	Alkaloids	Koenoline	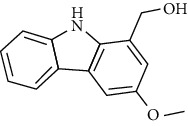	Cytotoxic	[23]
13	Leaves		Koenimbine	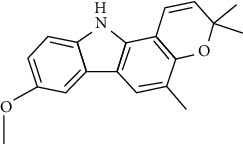	Antioxidant/antidiarrheal/anticancer	[7]
14	Roots		9-Formyl-3-methylcarbazole	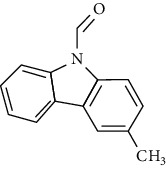	Anticancer	[7]
15	Leaves		O-methylmurrayamine A	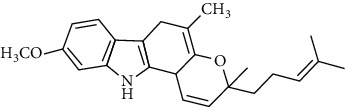	Antioxidant	[24]
16	Leaves	Essential oils	Linalool	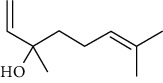	Antioxidant/antimicrobial	[25]
17			Elemol	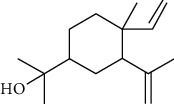		
18			Geranyl acetate	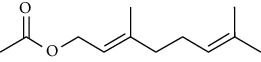		
19			Myrcene	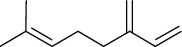		

20	Leaves	Essential oils	Allo-ocimene	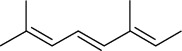	Antioxidant/antimicrobial	[25]
21			Α-terpinene	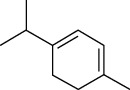		
22			(E)-*β*-ocimene	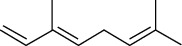		

23	Leaves	Flavonoids	Quercetin	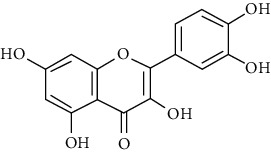	Antioxidant/anticancer	[26]
24			Catechin	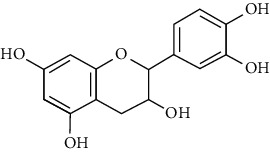		
25			Epicatechin	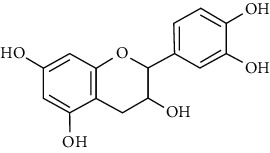		
26			Naringin	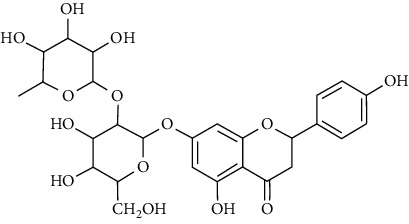	Antioxidant/anticancer	[26]
27			Myricetin	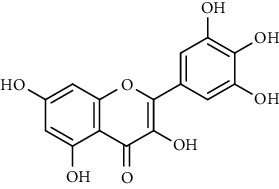		
28			Rutin	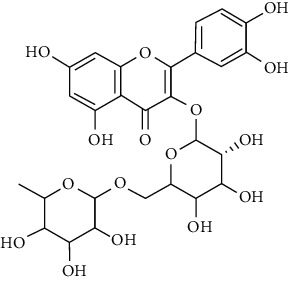		

29	Polyphenols		Gallic acid	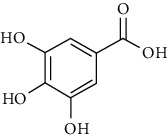	Antioxidant	[26]
30			Ferulic acid	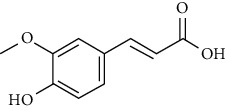		
31			Vanillic acid	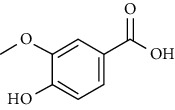		

**Table 3:** The major bioactive compounds of *M. minutum* and their pharmacological activities.

No	Plant part	Chemical type	Chemical	Structure	Activity	Ref
01	Leaves	Coumarins	Minutin A	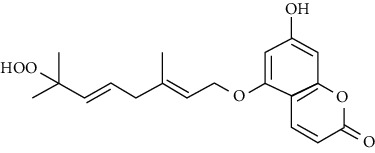	Anticancer/cytotoxic	[27]
02			Minutin B	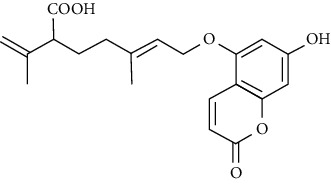		
03			Clauslactone E	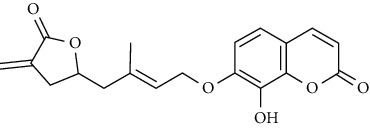		
04			8,4″- Dihydroxy-3″,4″-dihydrocapnolactone-2′,3′-diol	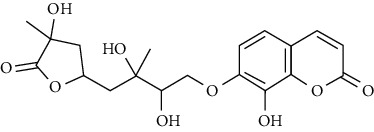		
05			8-Hydroxyisocapnolactone-2′,3′-diol	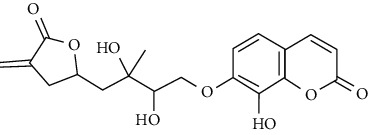		
06			8-Hydroxy-3″,4″- dihydrocapnolactone-2′,3′-diol	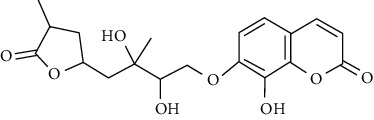		
07	Roots	Roots	Murralonginol isovalerate	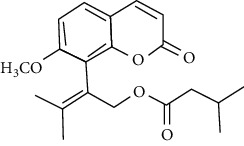	Cytotoxic	[28]
08			Osthol	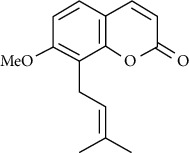		
09			Phebalosin	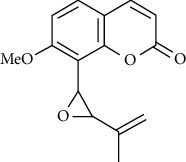		
10			Micromelin	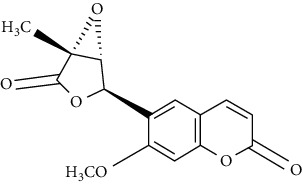		
11			Osthenon	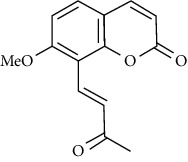	Cytotoxic	[28]
12			Umbelliferone	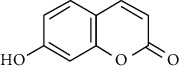		
13			Murracarpin	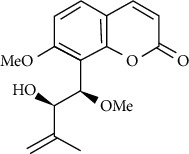		
14	Fruits		Murralonginol	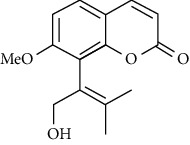	Cytotoxic/antitumor	[29]
15			Micromelin	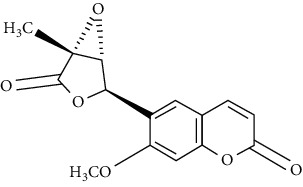		
16			Microminutin	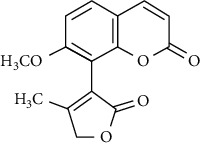		
17			Murrangatin	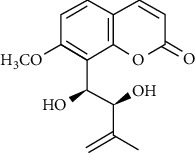		
18			Minumicrolin	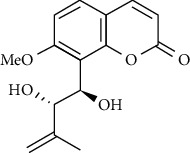		[29]
19			Scopoletin	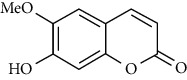		

20	Leaves	Essential oil	Bicyclogermacrene	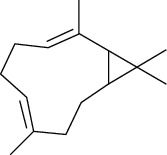	Antimicrobial	[30]
21			9-Epi-*β*-caryophyllene	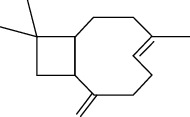		
22			Tricyclene	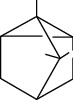		

**Table 4:** The major bioactive compounds of *C. indica* and their pharmacological activities.

No	Plant part	Chemical type	Chemical	Structure	Activity	Ref
01	Leaves	Essential oils	1-Menthone	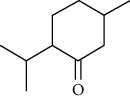	Antimicrobial	[31]
02			Terpinolene	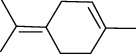	Antimicrobial/antioxidant/anticancer	[19]

03	Leaves	Essential oils	Terpinen-4-ol	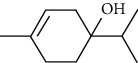	Antimicrobial	[14]
04			Sabinene	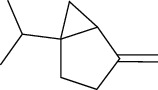		
05			*γ*-terpinene	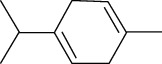		
06			*β*-phellandrene	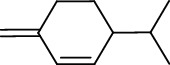		

**Table 5:** Microorganisms tested for the antimicrobial potential of *Murraya koenigii*^a^, *Micromelum minutum*^b^, and *Clausena indica*^c^.

		Species tested	Solvent	Ref
Leaves^a^	Well diffusion method	*Staphylococcus aureus*	Water, methanol	[76]
		*Salmonella typhi*		
		*Shigella* spp.		
		*Escherichia coli*		
		*Lactobacillus* spp.		

Leaves^a^	Well diffusion method	*Alternaria alternata*	Water	[78]
		*Aspergillus flavus*		
		*Aspergillus niger*		
		*Curvularia lunata*		
		*Fusarium moniliforme*		
		*F. solani*		
		*Helminthosporium sativum*		
		*Penicillium* spp.		

Leaves^a^	Well diffusion method	*Aspergillus niger*	Acetone, methanol, ethanol	[77]
		*Fusarium oxysporum Penicillium notatum Trichoderma viride*		
		*Bacillus cereus*		
		*Escherichia coli*		
		*Staphylococcus aureus Salmonella typhi*		

Seeds^a^	Disk diffusion method	*Klebsiella pneumoniae*	Acetone, chloroform, ethanol, methanol	[85]
		*Staphylococcus aureus Pseudomonas aeruginosa*		
		*Bacillus subtilis*		

Leaves and bark^a^	Agar cup-plate method	*Staphylococcus aureus*	Ethanol	[33]
		*Proteus vulgaris*		
		*Enterobacter aerogenes Lactobacillus* spp.		

Leaves and seed^a^	Disk diffusion method	*Klebsiella pneumoniae*	Acetone, chloroform, ethanol, methanol	[85]
		*Staphylococcus aureus Pseudomonas aeruginosa*		
		*Bacillus subtilis*		

Leaves and essential oils^a^	Well diffusion method	*Fusarium oxysporum Rhizoctonia solani*	Methanol, ethanol	[79]

Leaves^a^	Well diffusion method	*Escherichia coli*	Water, ethanol	[80]
		*Corynebacterium* spp. *Staphylococcus* spp.		
		*Bacillus* spp.		
		*Salmonella* spp.		

Leaves^a^	Well diffusion method	*Streptococcus mutans Streptococcus gordonii Pseudomonas aeruginosa*	Water, methanol, ethanol, n-hexane	[86]
		*Candida albicans*		

Leaves^a,b^	Well diffusion method	*Bacillus cereus*	Water, methanol, ethanol	[87]
		*Pseudomonas aeruginosa Staphylococcus aureus*		

Leaf^a^	Cup diffusion method	*Xanthomonas axonopodis pv. vesicatoria*	Chloroform, ethyl acetate, methanol	[88]
		*Xanthomonas campestris pv. campestris*		
		*Pseudomonas syringae*		
		*Aspergillus flavus*		
		*Fusarium verticillioides*		

Shoots^a^	Well diffusion method	*Escherichia coli*	n-Hexane, ethyl acetate, butanol, chloroform, water	[89]
		*Staphylococcus aureus Pseudomonas aeruginosa Salmonella typhi*		
		*Bacillus cereus*		
		*Klebsiella pneumoniae*		
		*Candida albicans*		

Essential oil^a^	Broth dilution method	*Staphylococcus aureus*		[90]
		*Escherichia faecalis*		
		*Escherichia coli*		
		*Pseudomonas aeruginosa*		
		*Candida albicans*		
		*Candida parapsilosis*		
		*Aspergillus fumigatus*		
		*Aspergillus niger*		

Leaf^a^	Well diffusion method	*Staphylococcus aureus Bacillus cereus*	Water	[91]
		*Micrococcus luteus*		
		*Escherichia coli*		
		*Klebsiella pneumoniae*		

Leaf^a^	Well diffusion method	*Staphylococcus aureus Bacillus cereus*	Ethanol, methanol, ethyl acetate, acetone, chloroform, petroleum ether, hexane, hot water	[92]
		*Micrococcus luteus Escherichia coli Pseudomonas aeruginosa Klebsiella pneumoniae*		
		*Aspergillus niger*		
		*Candida albicans*		
		*Candida tropicalis*		
		*Cryptococcus neoformans Candida* kefyr		

Leaf^a^	Disc diffusion method	*Staphylococcus aureus Escherichia coli*	Water	[93]
		*Staphylococcus epidermidis Pseudomonas aeruginosa Klebsiella pneumonia*		
		*Proteus mirabilis*		
		*Proteus vulgaris*		
		*Candida albicans*		
		*Aspergillus fumigatus*		

Leaves, stem, and root barks^b^	Disk diffusion method	*Staphylococcus aureus Escherichia coli*	Ethanol	[82]

Essential oil^b^	Well diffusion method	*Staphylococcus aureus*		[30]
		*Escherichia coli*		
		*Bacillus subtilis*		
		*Pseudomonas aeruginosa*		

Essential oil^c^	Broth diffusion method	*Escherichia coli*		[15]
		*Pseudomonas aeruginosa*		
		*Bacillus subtilis*		
		*Staphylococcus aureus*		
		*Aspergillus niger*		
		*Fusarium oxysporum*		
		*Saccharomyces cerevisiae*		
		*Candida albicans*		

Fruits^c^	Filter paper diffusion method, LB medium double dilution method	*Escherichia coli*		[84]
		*Staphylococcus aureus Bacillus subtilis*		
		*Mucor*		
		*Saccharomyces cerevisiae*		

Leaf essential oil^c^	Well diffusion method	*Bacillus cereus*		[14]
		*Salmonella typhi*		
		*Proteus vulgaris*		
		*Escherichia coli*		
		*Bacillus subtilis*		
		*Staphylococcus aureus*		
		*Klebsiella pneumoniae*		
		*Serratia marcescens*		

## Data Availability

All the data used to support the findings of this study are available from the corresponding author upon request.

## Supplementary Materials

Supplementary Materials Table S1: phytochemicals extracted from *Murraya koenigii.* Table S2: phytochemicals extracted from *Micromelum minutum.* Table S3: phytochemicals extracted from *Clausena indica*.
