# Supplementary material for: Nutritive Importance and Therapeutics Uses of Three Different Varieties (Murraya koenigii, Micromelum minutum, and Clausena indica) of Curry Leaves: An Updated Review
Source: Evid Based Complement Alternat Med. 2021 Oct 31;2021:5523252. doi: 10.1155/2021/5523252 (PMC8572594; doi:10.1155/2021/5523252)
Supplement: Supplementary Materials — Table S1: phytochemicals extracted from Murraya koenigii. Table S2: phytochemicals extracted from Micromelum minutum. Table S3: phytochemicals extracted from Clausena indica. [file 5523252.f1.docx]

Table S1:Phytochemicals extracted from *Murraya koenigii*

| No | Plant part | Chemical Type | Chemical | Structure | Ref |
| --- | --- | --- | --- | --- | --- |
| 1 | leaves | Alkaloids | Mahanimbicine |  | [1] |
| 2 | Bark/ Leaves /  Roots |  | Mahanimbilol /  Mahanimbinol |  | [2–4] |
| 3 | Leaves |  | Mahanimbidine |  | [5] |
| 4 |  |  | Cyclomahanimbidine |  | [6] |
| 5 |  |  | (+)-Isomahanimbine |  | [7] |
| 6 | Bark/ Leaves |  | Bismahanimboline |  | [2] |
| 7 | Leaves |  | Bicyclomahanimbicine |  | [6] |
| 8 | Bark/ Leaves |  | Bicyclomahanimbine |  | [2] |
| 9 | Bark/ Leaves |  | Mahaninebicine |  | [8] |
| 10 | Leaves |  | (R)-Mahanine |  | [1,8,9] |
| 11 | Leaves/ Bark/ Seeds |  | Isomahanine |  | [2] |
| 12 | Roots/ Stembark |  | Bismahanine |  | [4] |
| 13 | Bark/ Leaves |  | Bisisomahanine |  | [2] |
| 14 |  |  | Murrayafoline-A |  | [4] |
| 15 | Bark/ Leaves |  | Bismurrayafoline-E |  | [2] |
| 16 | Bark/ Leaves |  | Bispyrayafoline |  | [2,8] |
| 17 | Bark/Leaves |  | Euchrestine-A |  | [10] |
| 18 | Bark/ Leaves |  | Euchrestine-B |  | [2] |
| 19 | Stem |  | koenigine- quinone A |  | [11] |
| 20 | Stem |  | koenigine- quinone B |  | [11] |
| 21 | Leaves |  | Koenidine |  | [7,12] |
| 22 | Bark |  | Murrayazolidine |  | [13] |
| 23 | Bark/ Leaves |  | Murrayazolinol |  | [2] |
| 24 | Bark/ Leaves |  | Murrayamine-J |  | [2,14] |
| 25 | Bark/ Leaves |  | Murrayamine-B |  | [2] |
| 26 | Roots/ Stem |  | Mukoenine-A (Girinimbilol) |  | [3,4] |
| 27 | Roots/ Stem |  | Mukoenine-B |  | [3,4] |
| 28 | Roots |  | Mukoenine-C |  | [3,4] |
| 29 | Areal parts |  | Murrayadinal |  | [15] |
| 30 | Leaves |  | Murrayastine |  | [9] |
| 31 | Roots |  | Murrastifoline-A |  | [4] |
| 32 | Roots |  | Murrastifoline-F |  | [3] |
| 33 | Bark/ Leaves |  | Murrastanine-A |  | [2] |
| 34 | Bark/ Leaves |  | Murrastinine-A |  | [2] |
| 35 | Bark/ Leaves |  | Murrastinine-B |  | [2] |
| 36 | Bark/ Leaves |  | Murrastanine-C |  | [2] |
| 37 | Bark/ Leaves |  | Murrayatanine-A |  | [2] |
| 38 | Bark/ Leaves |  | Murrayaquinone-A |  | [2,4] |
| 39 | Bark/ Leaves |  | Murrayaquinone-B |  | [2] |
| 40 | Bark/ Leaves |  | Murrayakoeninol |  | [2] |
| 41 | Stem/ Leaves |  | Murrayakonine-A |  | [4,16] |
| 42 | Stem/ Leaves |  | Murrayakonine-B |  | [16] |
| 43 | Stem/ Leaves |  | Murrayakonine-C |  | [16] |
| 44 | Stem/ Leaves |  | Murrayakonine-D |  | [16] |
| 45 | Areal parts |  | Mukoenigatin |  | [15] |
| 46 | Stem/ Leaves |  | Mukolidine |  | [16] |
| 47 | Leaves |  | Mukonicine |  | [6] |
| 48 | Stem |  | Mukonal |  | [3] |
| 49 | Stem |  | Mukeic acid |  | [16] |
| 50 | Roots |  | Glycozoline |  | [4] |
| 51 | Root/ stem bark |  | Bis-2-hydroxy-3-methylcarbazole |  | [4] |
| 52 | Areal parts |  | Bikoeniquinonine |  | [15] |
| 53 | Root/ stembark |  | Bikoeniquinone A |  | [3,4] |
| 54 | Root/ stembark |  | Bismurrayaquinone A |  | [4] |
| 55 | Leaves |  | 8,8’’-Biskoenigine |  | [12] |
| 56 | Roots |  | Eustifoline A |  | [4] |
| 57 | Roots |  | Eustifoline C |  | [4] |
| 58 | Stem/ Leaves |  | 1-hydroxy-7-methoxy-8-(3-methylbut-2-en-1-yl)-9H-carbazole-3-carbaldehyde |  | [16] |
| 59 | Bark/ Leaves |  | 2-hydroxy-3-methylcarbazole |  | [2] |
| 60 | Roots |  | 9-carbethoxy-3-methylcarbazole |  | [17] |
| 61 | Bark |  | 3-formyl- carbazole |  | [4,13] |
| 62 | Bark |  | 3-formyl-8-methoxycarbazole |  | [13] |
| 63 | Stem bark |  | 3n-(1n-hydroxyethyl)-7-hydroxy-1-isobenzofuranone |  | [13] |
| 64 | Stem bark |  | 3,3’-[oxybis(methylene)]bis(9-methoxy- 9H-carbazole) |  | [13] |
| 65 | Bark |  | carbazole-3-carboxylic acid |  | [13] |
| 66 | Roots |  | 3-methyl-carbazole |  | [17] |
| 67 | Stem |  | 1. hydroxy -3- methyl carbazole |  | [3] |
| 68 | Leaves |  | O-methyl murrayamine |  | [8] |
| 69 | Leaves |  | O-methyl mahanine |  | [8] |
| 70 |  |  | 8,10′-[3,3′,11,11′-tetrahydro-9,9′-dihy- droxy-3,3′,5,8′-tetramethyl-3,3′-bis(4-methyl-3-pentenyl)]-bipyrano[3,2- a]carbazole |  | [8] |
| 71 | Whole plant | Alkenes | (3S,4E,6E,10R)-2,10-dihydroxy-2- hydroxy-2-methylethyl-6,10-di-methyl-4,6,11-sencolaninic-3-b- D-glucopyranoside. |  | [18] |
| 72 |  |  | (3R,5S,6E,8S,10E)-3,7,11-trimethyl-1,6,10-dodecatriene- 3,5,8-triol |  | [18] |
| 73 |  |  | (5S,6R,7S,8R)-5-amino-(2Z,4Z)-1,2,3-trihydroxybuta- 2,4-dienyloxy-pentane-6,7,8,9-tetraol |  | [18] |
| 74 |  |  | (3E,6S,7E,9R,10S,11S,17R)-octadeca-3,7-diene-6,9,10,11,17- pentaol |  | [18] |
| 75 |  |  | (2E,6R)-2,6-dimethyl- 2,7-octadiene-1,6-diol |  | [18] |
| 76 |  |  | (6R,7E,9S,10R)-6,9,10-trihydroxy-7- octadecenoic acid |  | [18] |
| 77 |  |  | capsianoside V |  | [18] |
| 78 |  |  | (9S,10R,11R,12Z,15Z)-9,10,11-trihydroxy-octadeca-12,15-dienoic acid |  | [18] |
| 79 |  |  | (8R,9R,10S,6Z)-trihydroxyoctadec-6-enoic acid |  | [18] |
| 80 |  |  | (9S,10R,11E,13S)-9,10,13-trihydroxyoctadec-11-enoic acid |  | [18] |
| 81 |  |  | (8S,9R,10E,12S,14Z)-heptadeca-10,14-diene-1,8,9,12- tetraol |  | [18] |
| 82 | Bark | Sterol | β-sitosterol |  | [13] |
| 83 |  |  | Stigmasterol |  | [13] |
| 84 |  |  | Osthol |  | [19] |
| 85 |  |  | Umbelliferone |  | [19] |
| 86 |  | Flavonoids | Apigenin |  | [20] |
| 87 |  |  | Kaempferol |  |  |
| 88 | Leaves | Terpenoids | (3R,5S,6R)-3,5,6-trihydroxy1,1,5-trimethylcyclohexyl-8-butyn-9-one |  | [21] |
| 89 |  |  | (3S,5R,6R,8E)-3,5,6-trihydroxy-1,1,5-trimethylcyclohexyl-8-buten-9-one |  |  |
| 90 |  |  | (8E,9R)-ethyl-7-(3S,5R,6S)-3,6- dihydroxy-1,1,5-trimethylcyclohexyl-9-hydroxybut-8-enoate |  |  |
| 91 |  |  | (3R)-3-O-β-Dglucoside-6′-D-apiose-β-ionone. |  |  |
| 92 |  |  | (R)-(−)-dehydrovomifoliol |  | [21] |
| 93 |  |  | Blumenol A |  |  |
| 94 |  |  | Blumenol C |  |  |
| 95 |  |  | Icariside B1 |  |  |
| 96 |  |  | (4R)-4-hydroxy-2,6,6-trimethyl-1-cyclohexene-1-methanol |  |  |
| 97 |  |  | (8E)-3(R)-O-β-D-glucopyranosyloxy-1,5,5-trimethyl-7-cyclohexen-8-buten-7-one |  |  |
| 98 |  |  | 3β-hydroxy5α,6α-epoxy-7-megastigmen-9-one |  |  |
| 99 |  |  | 3-O-β-D-glucopyranosyloxy-5α,6α-epoxy-7-megastigmen-9-one |  |  |
| 100 |  |  | Loliolide |  |  |
| 101 |  |  | 8 (−)-epiloliolide |  |  |
| 102 |  |  | (3R,6R,7E)-3-hydroxy-α-ionone |  |  |
| 103 |  |  | Cinnamic acid |  |  |
| 104 | Leaves | Essential -oils | Allyl(methoxy)dimethyl  silane |  | [22] |
| 105 |  |  | Caryophyllene oxide |  |  |
| 106 |  |  | Caryophyllene |  |  |
| 107 |  |  | Camphene |  |  |
| 108 |  |  | Cubenol |  |  |
| 109 |  |  | Cis-Piperitol |  |  |
| 110 |  |  | Cis-sabinenehydrate |  |  |
| 111 |  |  | Eucalyptol |  |  |
| 112 |  |  | Eudesma-4(14),11-diene |  |  |
| 113 |  |  | Isobornyl acetate |  |  |
| 114 |  |  | Juniper camphor |  |  |
| 115 |  |  | Limonene |  |  |
| 116 |  |  | m-Cymene |  |  |
| 117 |  |  | Naphthalene |  |  |
| 118 |  |  | Neryl propionate |  |  |
| 119 |  |  | Nerolidyl acetate |  |  |
| 120 |  |  | Phytol |  |  |
| 121 |  |  | Terpinyl acetate |  |  |
| 122 |  |  | α-caryophyllene |  |  |
| 123 |  |  | α-thujene |  |  |
| 124 |  |  | α-Phellandrene |  |  |
| 125 |  |  | α-Cadinol |  |  |
| 126 |  |  | Myrcene |  |  |
| 127 |  |  | β-elemene |  |  |
| 128 |  |  | β-Phellandrene |  |  |
| 129 |  |  | γ-terpinene |  |  |
| 130 |  |  | δ-Elemene |  |  |
| 131 |  |  | δ-Cadinene |  |  |
| 132 |  |  | 1-Chloroheptacosane |  |  |
| 133 |  |  | 1,4-Methanoazulen-9-ol, decahydro-  1,5,5,8a-tetramethyl |  |  |
| 134 |  |  | 2(1H)-Naphthalenone, 4a,5,6,7,8,8ahexahydro-  4a,8a-dimethyl |  |  |
| 135 |  |  | 3-Phenylbutyrophenone |  |  |
| 136 |  |  | 3-carene |  |  |
| 137 |  |  | 4-Terpineol |  |  |
| 138 |  |  | 12-Oxabicyclo[9.1.0]dodeca-3,7-diene,  1,5,5,8-tetramethyl |  |  |
| 139 | Leaves | Rutinocides | 4-O-β-D-rutinosyl-3-methoxyphenyl-1-propanone |  | [21] |
| 140 |  |  | 1-O-β-D-rutinosyl2(R)-ethyl-1-pentanol |  |  |
| 141 |  |  | 8-phenylethyl-O-β-D-rutinoside |  |  |

Table S2: Phytochemicals extracted from *Micromelu mminutum*

| No | Plant part | Chemical type | chemical |  | References |
| --- | --- | --- | --- | --- | --- |
| 1 | Leaves | coumarins | Dihydromicromelin B |  | [23] |
| 2 |  |  | Micromarin-A |  | [24] |
| 3 |  |  | Micromarin-B |  |  |
| 4 |  |  | Micromarin-C |  |  |
| 5 |  |  | Micromarin-F |  |  |
| 6 |  |  | Micromarin-G  Micromarin-H |  |  |
| 7 |  |  |  |  |  |
| 8 |  |  | Micromelumin |  | [23] |
| 9 |  |  | Microminutinin |  | [24] |
| 10 |  |  | Micropubescin |  | [25] |
| 11 |  |  | Minutuminolate |  | [26] |
| 12 |  |  | Mupanidin |  | [25] |
| 13 |  |  | Murrangatin acetate |  | [26] |
| 14 |  |  | Murralongin |  | [25] |
| 15 |  |  | Stigmasterol |  | [27,28] |
| 16 |  |  | 3”,4”-dihydrocapnolactone |  | [27] |
| 17 |  |  | 2’,3’-epoxyisocapnolactone |  | [27] |
| 18 | Fruits |  | 6-methoxy microminutinin |  | [24] |
| 19 |  |  | 8-hydroxyisocapnolactone-  2’,3’-diol |  | [27] |
| 20 | Leaves |  | 8-methoxycapnolactone |  | [29] |
| 21 | Roots | Alkaloids | Flindersine |  | [25] |
| 22 |  | Flavonoids | 5,7-dihydroxy-  3,4’,6,8-tetramethoxyflavone |  | [25] |
| 23 |  |  | Citronellyl formate |  | [28] |
| 24 | Leaves |  | Caryophyllene oxide |  | [28] |
| 25 |  |  | Epi-α-cadinol |  | [28] |
| 26 |  |  | Epi-α-muurolol  (T-muurolol) |  | [28] |
| 27 |  |  | Germacrene A |  | [28] |
| 28 |  |  | Germacrene D |  | [28] |
| 29 | Leaves |  | Globulol |  | [28] |
| 30 |  |  | Limonene |  | [28] |
| 31 |  |  | Longiborneol acetate |  | [28] |
| 32 |  |  | Myrcene |  | [30] |
| 33 |  |  | Safrole |  | [30] |
| 34 |  |  | Tetracosane |  | [30] |
| 35 | Leaves |  | Terpinen-4-ol |  | [30] |
| 36 |  |  | Undecanal |  | [28] |
| 37 |  |  | α –Cadinol |  | [28] |
| 38 |  |  | α – Humulene |  | [28] |
| 39 |  |  | α – Selinene |  |  |
| 40 |  |  | α –Terpinene |  |  |
| 41 |  |  | α – Terpineol |  | [28] |
| 42 |  |  | β – Bisabolenol |  | [28] |
| 43 |  |  | β – Caryophyllene |  | [30] |
| 44 |  |  | β –Elemene |  | [28] |
| 45 |  |  | β – Pinene |  | [28] |
| 46 |  |  | β – Oplopenone |  | [28] |
| 47 |  |  | β - Selinene |  | [28] |
| 48 |  |  | δ - Cadinene |  | [28] |
| 49 |  |  | n – Decanal |  | [28] |
| 50 |  |  | (Z) – Nerolidol |  | [28] |
| 51 |  |  | 1,8- Cineole |  | [30] |
| 52 |  | Terpenes | 5(6)-gluten-3-one |  | [29] |
| 53 |  |  | 5(6)-gluten-3α-ol |  | [29] |

Table S3: Phytochemicals extracted from *Clausena indica*

| No | Plant part | Chemical type | Chemical | Structure | Ref |
| --- | --- | --- | --- | --- | --- |
| 1 | Roots | Carbazole alkaloids | 6-methoxyheptaphylline |  | [31–33] |
| 2 |  |  | Indizoline |  |  |
| 3 |  |  | Lansine |  | [32] |
| 4 | Roots | Coumarins | Imperatorin |  | [33] |
| 5 |  |  | Phellopterin |  |  |
| 6 | Roots/ leaves |  | Chalepensin |  |  |
| 7 |  |  | Chalepin |  |  |
| 8 | Roots |  | Clausindine |  |  |
| 9 | Leaves/Branches | Essential oils | Benzaldehyde |  | [34] |
| 10 |  |  | Benzylbenzoate |  |  |
| 11 |  |  | Bicyclogermacrene |  |  |
| 12 |  |  | Caryophyllene oxide |  |  |
| 13 |  |  | Carotol |  |  |
| 14 |  |  | Camphene |  |  |
| 15 |  |  | Carvacrol |  |  |
| 16 |  |  | Cis-verbenol |  |  |
| 17 |  |  | *Cis*-pinocamphone |  |  |
| 18 |  |  | Elemicin |  |  |
| 19 |  |  | *Endo*-Fenchyl acetate |  |  |
| 20 |  |  | Eugenol |  |  |
| 21 |  |  | Humulene epoxide II |  |  |
| 22 |  |  | Methyl euginole |  |  |
| 23 |  |  | Pentadecanal |  |  |
| 24 |  |  | Isodaucene |  |  |
| 25 |  |  | γ-muurolene |  |  |
| 26 |  |  | Thymol |  |  |
| 27 |  |  | Verbenone |  |  |
| 28 |  |  | α-Thujene |  |  |
| 29 |  |  | α-Humulene |  |  |
| 30 |  |  | α-Fenchene |  |  |
| 31 |  |  | α-Pinene |  |  |
| 32 |  |  | α-phellandrene |  |  |
| 33 |  |  | α-terpinene |  |  |
| 34 |  |  | α-terpineol |  |  |
| 35 |  |  | β-pinene |  |  |
| 36 |  |  | γ-terpineol |  |  |
| 37 |  |  | P-cymene |  |  |
| 38 |  |  | p-mentha-1,5-dien-8-ol |  |  |
| 39 |  |  | Limonene |  |  |
| 40 |  |  | Linalool |  |  |
| 41 |  |  | Myristicin |  |  |
| 42 |  |  | Myrcene |  |  |
| 43 |  |  | o-cymene |  | [35] |
| 44 |  |  | P-cymen-8-ol |  | [35,36] |
| 45 |  |  | Piperitenone |  |  |
| 46 |  |  | Spathulenol |  |  |
| 47 |  |  | p-menth-2-en-1-ol |  |  |
| 48 |  |  | Terpinolene |  |  |
| 49 |  |  | Tricyclene |  |  |
| 50 |  |  | (Z)-β-ocimene |  |  |
| 51 |  |  | (E)-β-ocimene |  |  |
| 52 |  |  | (E-E)-α-Farnesene |  | [37] |
| 53 |  |  | (E)-geranyl-acetone |  |  |
| 54 |  |  | Nerolidol |  | [37] |
| 55 |  |  | (E)-2-Hexenal |  | [35] |
| 56 |  |  | δ-2-carene |  | [37] |
| 57 |  |  | δ-3-carene |  |  |
| 58 |  |  | 1,3,8-p-menthatriene |  |  |
| 59 |  |  | sabinene hydrate |  | [38] |
| 60 |  |  | 4-carene |  |  |
| 61 |  |  | 2,3-dehydro-1,8-cineole |  | [35,39] |
| 62 |  |  | 3Z-hexenyl benzoate |  |  |
| 63 |  |  | β-Bourbonene |  |  |
| 64 |  |  | β-bisabolene |  |  |
| 65 |  |  | Fenchyl acetate |  | [35] |
| 66 | Leaves/branchs |  | 2,6-bis(1,1-dimethylethyl)-4- methylphenol |  |  |
| 67 |  |  | 6-camphenone |  |  |
| 68 | Leaves | Amides | Balasubramide |  | [40] |
| 69 |  |  | Prebalamide |  |  |
| 70 |  |  | Madugin |  |  |
| 71 |  |  | Methylmadugin |  |  |

References

[1] Y. Tachibana, H. Kikuzaki, N.H. Lajis, N. Nakatani, Antioxidative activity of carbazoles from Murraya koenigii leaves, J. Agric. Food Chem. 49 (2001) 5589–5594.

[2] S.P. Tan, A.M. Ali, M.A. Nafiah, K. Awang, K. Ahmad, Isolation and cytotoxic investigation of new carbazole alkaloids from Murraya koenigii (Linn.) Spreng, Elsevier Ltd, 2015.

[3] D.K. Gahlawat, S. Jakhar, P. Dahiya, Murraya koenigii (L.) Spreng: an ethnobotanical, phytochemical and pharmacological review, J. Pharmacogn. Phytochem. 3 (2014) 109–119.

[4] C. Ito, Y. Thoyama, M. Omura, I. Kajiura, H. Furukawa, Alkaloidal Constituents of Murraya koenigii. Isolation and Structural Elucidation of Noval Binary Carbazolequinones ans Carbazole Alkaloids, Chem. Pharm. Bull. 41 (1993) 2096–2100.

[5] S.P. Kureel, R.S. Kapil, S.P. Popli, Terpenoid alkaloids from Murraya koenigii spreng.-II.: The constitution of cyclomahanimbine, bicyclomahanimbine, and mahanimbidine., Tetrahedron Lett. 10 (1969) 3857–3862.

[6] A. Ismail, B. Noolu, R. Gogulothu, S. Perugu, A. Rajanna, S.K. Babu, Cytotoxicity and Proteasome Inhibition by Alkaloid Extract from Murraya koenigii Leaves in Breast Cancer Cells - Molecular Docking Studies, J. Med. Food. 19 (2016) 1155–1165.

[7] N.S. Narasimhan, M. V Paradkar, V.P. Chitguppi, S.L. Kelkar, Alkaloids of Murraya koenigii: structures of mahanimbine, koenimbine, (-)-mahanine, koenine, koenigine, koenidine & (+)-isomahanimbine, Indian J. Chem. 13 (1975) 993–999.

[8] Y. Tachibana, H. Kikuzaki, N.H. Lajis, N. Nakatani, Comparison of Antioxidative Properties of Carbazole Alkaloids from Murraya koenigii Leaves, J. Agric. Food Chem. 51 (2003) 6461–6467.

[9] S.K. Samanta, R. Kandimalla, B. Gogoi, K.N. Dutta, P. Choudhury, P.K. Deb, R. Devi, B.C. Pal, N.C. Talukdar, Phytochemical portfolio and anticancer activity of Murraya koenigii and its primary active component, mahanine, Pharmacol. Res. 129 (2018) 227–236.

[10] I.J. Chibueze, I. V Emenike, Murraya koenigi-a boon in different pathological conditions, Univers. J. Pharm. Cond. 1 (2016) 61–68.

[11] C. Saha, B.K. Chowdhury, Carbazoloquinones from Murraya koenigii, Phytochemistry. 48 (1998) 363–366.

[12] O.P.S. Patel, A. Mishra, R. Maurya, D. Saini, J. Pandey, I. Taneja, K.S.R. Raju, S. Kanojiya, S.K. Shukla, M.N. Srivastava, M. Wahajuddin, A.K. Tamrakar, A.K. Srivastava, P.P. Yadav, Naturally Occurring Carbazole Alkaloids from Murraya koenigii as Potential Antidiabetic Agents, J. Nat. Prod. 79 (2016) 1276–1284.

[13] M.M. Rahman, A.I. Gray, A benzoisofuranone derivative and carbazole alkaloids from Murraya koenigii and their antimicrobial activity, Phytochemistry. 66 (2005) 1601–1606.

[14] Y. Nalli, V. Khajuria, S. Gupta, P. Arora, S. Riyaz-Ul-Hassan, Z. Ahmed, A. Ali, Four new carbazole alkaloids from Murraya koenigii that display anti-inflammatory and anti-microbial activities, Org. Biomol. Chem. 14 (2016) 3322–3332.

[15] S. Naz, S. Saied, A. Ahmed, S.M. Shahid, Three new carbazole alkaloids and biological activities of Murraya koenigii, J. Asian Nat. Prod. Res. 17 (2015) 7–13.

[16] B.K. Chowdhury, D.B. Chakraborty, Mokoeic acid-first carbazole carboxylic acid from plant sources, Chem. Ind. 17 (1969) 549.

[17] M.N. Chakrabarty, S.C. A..C Khasnobis, M., Y. Konda, Y. Harigaya, Komiyama., Carbazole Alkaloids from Murraya Koenigii, Phytochemistry. 46 (1997) 751–755.

[18] Q.G. Ma, K. Xu, Z.P. Sang, R.R. Wei, W.M. Liu, Y.L. Su, J.B. Yang, A.G. Wang, T.F. Ji, L.J. Li, Alkenes with antioxidative activities from Murraya koenigii (L.) Spreng, Bioorganic Med. Chem. Lett. 26 (2016) 799–803.

[19] V. Jain, M. Momin, K. Laddha, Murraya koenigii: An Updated Review, Int. J. Ayurvedic Herb. Med. 2 (2012) 607–627.

[20] B. Noolu, R. Gogulothu, M. Bhat, S. S.Y.H. Qadri, V. Sudhakar Reddy, G. Bhanuprakash Reddy, A. Ismail, In Vivo Inhibition of Proteasome Activity and Tumour Growth by Murraya koenigii Leaf Extract in Breast Cancer Xenografts and by Its Active Flavonoids in Breast Cancer Cells, Anticancer. Agents Med. Chem. 16 (2016) 1605–1614.

[21] Q.G. Ma, Y.G. Wang, W.M. Liu, R.R. Wei, J.B. Yang, A.G. Wang, T.F. Ji, J. Tian, Y.L. Su, Hepatoprotective sesquiterpenes and rutinosides from Murraya koenigii (L.) spreng, J. Agric. Food Chem. 62 (2014) 4145–4151.

[22] J.U. Chowdhury, M.N.I. Bhuiyan, M. Yusuf, Chemical composition of the leaf essential oils of Murraya koenigii (L.) Spreng and Murraya paniculata (L.) Jack, Bangladesh J. Pharmacol. 3 (2008) 59–63.

[23] S. Das, R.H. Baruah, R.P. Sharma, J. Barua, P. Kulanthaivel, W. Herz, 7-Methoxycoumarins From Micromelum Minutum, Phytochemistry. 23 (1984) 2317–2321.

[24] I. Chihiro, T. Otsuka, N. Ruangrungsi, H. Furukawa, Chemical Constituents of Micromelum minutum. Isolation and Structural Elucidation of New Coumarins., Chem Pharm Bull. 48 (2000) 334–338.

[25] Y.C. Kong, P.P.H. But, K. hung Ng, Q. Li, K.F. Cheng, P.G. Waterman, Micromelum: a key genus in the chemosystematics of the Clauseneae, Biochem. Syst. Ecol. 16 (1988) 485–489.

[26] R. Lekphrom, K. Kanokmedhakul, W. Sangsopha, S. Kanokmedhakul, A new coumarin from the roots of Micromelum minutum, Nat. Prod. Res. 30 (2016) 2383–2388.

[27] N.B. Alitheen, S.K. Yeap, Cytotoxic effect of 2 ’, 3 ’ -epoxy isocapnolactone and 8- hydroxyisocapnolactone-2 ’ 3 ’ -diol isolated from Micromelum minutum ( G . Forst .) Wight and Arn . in human T-lymphocyte leukemia CEM-SS cells, African J. Biotechnol. 8 (2009) 4632–4641.

[28] N. Kamkaen, N. Ruangrangsi, Chemical compositions and antimicrobial activities of essential oils from Micromelum minutum, Thai J Heal. Res. 17 (2003) 148–153.

[29] R.A. Susidarti, M. Rahmani, A.M. Ali, M.A. Sukari, H.B.M. Ismail, J. Kulip, P.G. Waterman, 8-Methoxycapnolactone and stigmasterol From Micromelum minutum, Maj. Farm. Indones. 18 (2007) 105–109.

[30] P.A. Paranagama, J.J. Gunasekera, the efficacy of the essential oils of sri lankan cinnamomum zeylanicum fruit and micromelum minutum leaf against callosobruchus maculatus (f.) (coleoptera: Bruchidae), J. Essent. Oil Res. 23 (2011) 75–82.

[31] B.S. Loski, V.-N. Kamat, D.H. Gawad, Clausindine, a Novel Cyclopropylcoumarin, (1974) 1562–1564.

[32] C. Chaichantipyuth, S. Pummangura, K. Naowsaran, D. Thanyavuthi, J.E. Anderson, J.L. McLaughlin, Two New Bioactive Carbazole Alkaloids from the Root Bark of Clausena Harmandiana, J. Nat. Prod. 51 (1988) 1285–1288.

[33] N. Quan, T. Dang Xuan, Anh, Tran, H.D. Quan, N.V.; Xuan, T.D.; Anh, L.H.; Tran, Bio-guided isolation of prospective bioactive constituents from roots of Clausena indica (Dalzell) oliv., Molecules. 24 (2019) 4442.

[34] P.T.M. Diep, A.M. Pawlowska, P.L. Cioni, C. Van Minh, L.M. Huong, A. Braca, Chemical Composition and Antimicrobial Activity of Clausena Indica (Dalz) Oliv. (Rutaceae) Essential Oil from Vietnam, Nat. Prod. Commun. 4 (2009) 869–872.

[35] A.B. Pham Thi Minh Diep, Agata Maria Pawlowska, Pier Luigi Cioni, Chau Van Minh, Le Mai Huong, Natural Product Communications Chemical Composition and Antimicrobial Activity of, Nat. Prod. Commun. 4 (2009) 8–11.

[36] J.A. John, S.R.R. Kurup, N.S. Pradeep, B. Sabulal, A. John, R. Kurup, N.S. Pradeep, B. Sabulal, Chemical composition and antibacterial activity of the leaf oil of clausena indica from south india, J. Essent. Oil-Bearing Plants. 14 (2011) 776–781.

[37] J.A. John, S.R.R. Kurup, N.S. Pradeep, B. Sabulal, Chemical composition and antibacterial activity of the leaf oil of clausena indica from south india, J. Essent. Oil-Bearing Plants. 14 (2011) 776–781.

[38] T.H. Thai, O. Bazzali, T.M. Hoi, N.T. Hien, N.V. Hung, F. Tomi, J. Casanova, A. Bighelli, Chemical composition of the essential oils from vietnamese clausena indica and c. anisum-olens, Nat. Prod. Commun. 9 (2014) 1531–1534.

[39] T.H. Thai, O. Bazzali, T.M. Hoi, N.T. Hien, N.V. Hung, F.F. Tomi, J. Casanova, A. Bighelli, T. Thaia, O. Bazzali, T.M. Hoi, N. Hiền, N.V. Hung, F.F. Tomi, J. Casanova, A. Bighelli, Chemical Composition of the Essential Oils from Vietnamese Clausena indica and C. anisum-olens, Nat. Prod. Commun. 9 (2014) 1531–1534.

[40] B. Riemer, O. Hofer, H. Greger, Tryptamine Derived Amides from Clausena indica, Phytochemistry. 45 (1997) 337–341.
